# Update on Foregut Molecular Embryology and Role of Regenerative Medicine Therapies

**DOI:** 10.3389/fped.2017.00091

**Published:** 2017-04-28

**Authors:** Silvia Perin, Conor J. McCann, Osvaldo Borrelli, Paolo De Coppi, Nikhil Thapar

**Affiliations:** ^1^Stem Cells and Regenerative Medicine, UCL Great Ormond Street Institute of Child Health, London, UK; ^2^Neurogastroenterology and Motility Unit, Department of Gastroenterology, Great Ormond Street Hospital NHS Foundation Trust, London, UK; ^3^Specialist Neonatal and Paediatric Surgery (SNAPS) Department, Great Ormond Street Hospital NHS Foundation Trust, London, UK

**Keywords:** esophageal atresia, tracheo-esophageal fistula, foregut development, stem cell, tissue engineering, enteric nervous system

## Abstract

Esophageal atresia (OA) represents one of the commonest and most severe developmental disorders of the foregut, the most proximal segment of the gastrointestinal (GI) tract (esophagus and stomach) in embryological terms. Of intrigue is the common origin from this foregut of two very diverse functional entities, the digestive and respiratory systems. OA appears to result from incomplete separation of the ventral and dorsal parts of the foregut during development, resulting in disruption of esophageal anatomy and frequent association with tracheo-oesophageal fistula. Not surprisingly, and likely inherent to OA, are associated abnormalities in components of the enteric neuromusculature and ultimately loss of esophageal functional integrity. An appreciation of such developmental processes and associated defects has not only enhanced our understanding of the etiopathogenesis underlying such devastating defects but also highlighted the potential of novel corrective therapies. There has been considerable progress in the identification and propagation of neural crest stem cells from the GI tract itself or derived from pluripotent cells. Such cells have been successfully transplanted into models of enteric neuropathy confirming their ability to functionally integrate and replenish missing or defective enteric nerves. Combinatorial approaches in tissue engineering hold significant promise for the generation of organ-specific scaffolds such as the esophagus with current initiatives directed toward their cellularization to facilitate optimal function. This chapter outlines the most current understanding of the molecular embryology underlying foregut development and OA, and also explores the promise of regenerative medicine.

## Introduction

OA affects approximately 1 in 3,500 live births (1). Surgical correction aims at reconstituting gut continuity and disrupting the connection between the digestive and respiratory systems but despite considerable surgical expertise, including the introduction of minimally invasive approaches, the prognosis remains guarded and quality of life throughout childhood and adolescence poor. Affected children and adults continue to suffer from severe gastroesophageal reflux (GER), esophagitis, dysphagia, and esophageal dysmotility as well as poor weight gain together with chronic respiratory infections, tracheomalacia, and decreased exercise tolerance (2, 3). Although definitive surgery is carried out early in life, children with OA often require further interventions such as esophageal dilatations.

Surgically, OA is typically classified in two main groups according to the distance of separation between the two esophageal pouches: long gap OA and non-long gap OA. The most used definition of long gap OA is a gap greater than two to four vertebral bodies or 4–6 cm in length, although others have defined it as the inability of joining the esophagus at the first surgery with the result that there has been no unanimous definition for the two groups (4). In the current issue, the International Network of Esophageal Atresia has proposed that any OA that has no intra-abdominal air should be considered as long-gap (see the article by Van Der Zee et al.).

While other classifications are available and discussed in other articles of this special edition, the authors believe that distinguishing long gap OA from other forms is therapeutically important. In this group of patients, repair can present a significant surgical challenge and an esophageal replacement is often used. This can include gastric transposition (often called “gastric pull-up”) (5), colonic (6), or jejunal interposition (7). Such interventions are generally reliant on the position and length of the remaining native esophagus. During gastric pull-up procedures, the entire stomach including its vascular supply is moved into the mediastinum and a pyloroplasty is usually performed in an attempt to avoid delayed gastric emptying (8). An esophageal substitute can also be created from the larger curvature of the stomach, without moving the stomach itself [gastric tube esophagoplasty (9)]. In other cases, either jejunum or colon is used as substitute, with sections of these organs moved together with their own vasculature (6–8). More recently, closure of the gap by mechanical lengthening *via* external traction has been attempted by several surgeons (10–12), with Khan et al. reporting preservation, in terms of thickness, of the mural layers of the esophagus after this treatment (13).

Despite these efforts a definitive therapy for OA has yet to be developed. Such efforts have been halted somewhat by a failure in determining the precise etiopathogenesis of OA in human patients. Even with advances in genetic diagnostics, the genetics of OA represents a challenge, as the condition is frequently associated with malformations in other organs, especially congenital defects of the heart and of other endodermal organs. For instance, VACTERL syndrome is characterized by the involvement of defects in at least three body systems from the vertebral, anorectal, cardiovascular, tracheal, esophageal, renal, and limb systems. Tracheo-esophageal fistula (TOF) has been reported to be variably associated with this syndrome in between 50 and 80% of cases (14–16). There is, however, emerging evidence of an important role for genetic factors in the molecular specification of foregut development. Significant evidence has been garnered from multiple transgenic animal models, which are beginning to shed light on possible dysfunctional mechanisms resulting in OA ± associated TOF, which may have translational consequences for clinical diagnostics in human OA.

## Gross Development of the Foregut: Models for the Pathogenesis of OA/TOF

The gastrointestinal (GI) tract is a complex physiological system comprising the hollow organs of the digestive system (pharynx, esophagus, stomach, intestine, and colon), usually termed the “gut” and the GI tract derivatives (thyroid, thymus, parathyroid, lungs, liver, and pancreas). Throughout the GI tract, each region exists as a sophisticated multi-layered system consisting of a mucosal layer, neural plexuses, and a number of muscle layers. Developmentally, all three germ layers participate in the formation of the gut. The endoderm and mesoderm form the epithelial layer and muscle layers, respectively, with the ectoderm forming the various neural plexuses present throughout the GI tract termed “the enteric nervous system.” Initially, the embryonic gut develops as a result of cephalocaudal and lateral embryo folding and incorporation of the endoderm-lined yolk sac. This leads to the formation of two blind-ending endodermal invaginations at the anterior and posterior ends of the embryo, which fuse to give rise to the primitive gut. This primitive gut structure subsequently undergoes significant patterning along the anterior–posterior axis and is delineated into three main areas: the foregut (esophagus and stomach), midgut (small intestine), and hindgut (colon) (17). Anatomically, the foregut can further be divided into two portions, the anterior and posterior foregut, with the former giving rise to the esophagus, trachea, and lungs and the latter to the stomach, pancreas, and liver.

**Figure 1:**
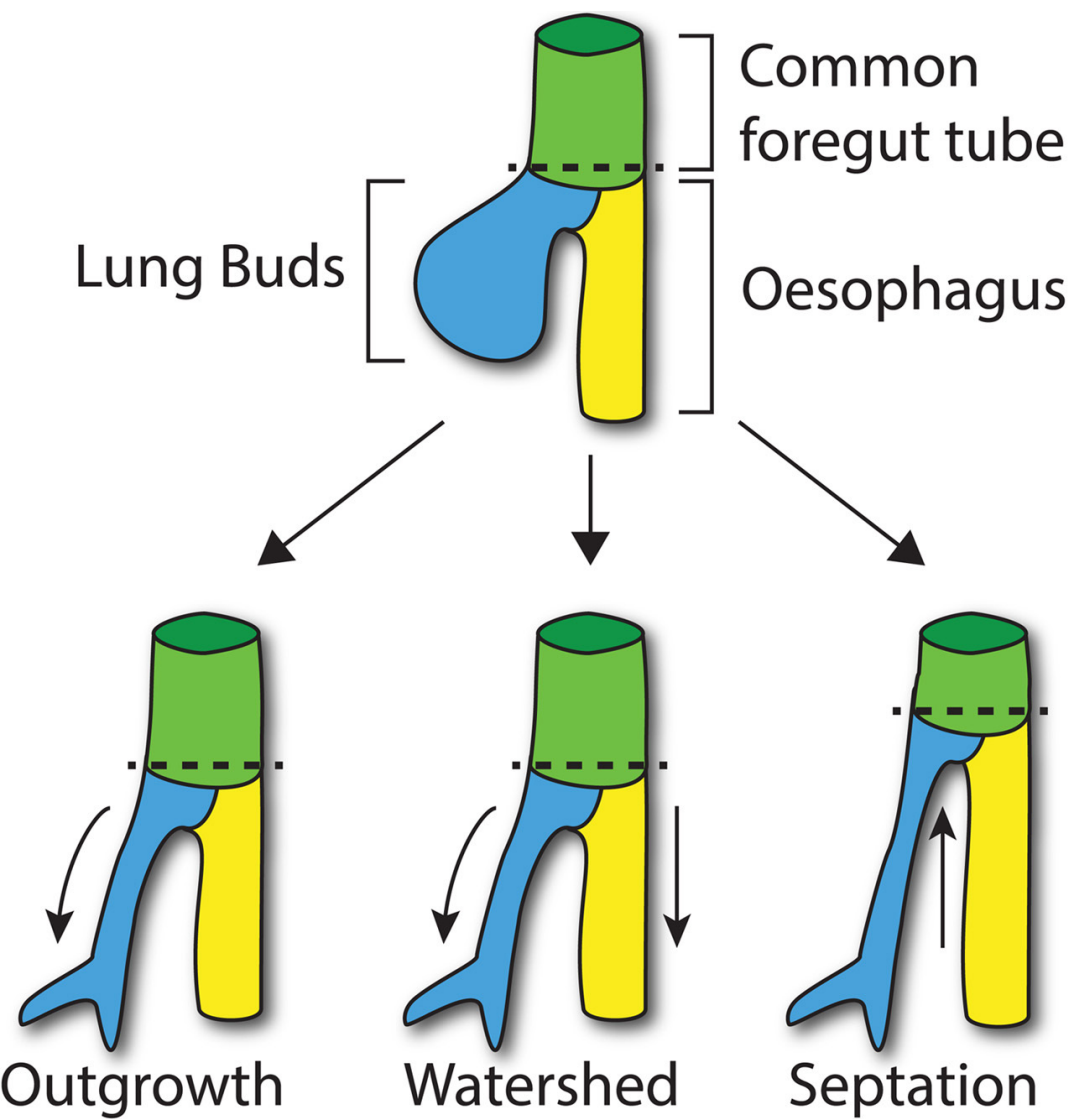
**Compartmentalization of the foregut**. At E9.5 in the mouse, the lung buds start to arise from the common foregut tube (dashed line; top). According to the Outgrowth model (bottom left), the trachea extends from the foregut tube at the level where lung buds develop (curved arrow). The Watershed model suggests that both developing trachea and esophagus elongate (arrows) from the diverging point (dashed line; middle). According to the Septation model (bottom right), a septum is formed from lateral ridges of mesenchyme, which moves up along the longitudinal axis of the common foregut tube separating the trachea and esophagus (arrow).

Of particular interest to the development of the foregut is the common origin of both the digestive and respiratory systems. Despite their differing function, the digestive and respiratory systems share a common embryonic origin, deriving from the developing anterior foregut. In mouse, between embryonic (E) days 9.5 and 11.5 (equivalent to weeks 4–6 in human gestation), a compartmentalization process takes place with the formation of the respiratory diverticulum (lung buds) from the ventral anterior foregut endoderm and the gradual separation of the ventral respiratory diverticulum from the dorsal anterior foregut by the esophagotracheal septum (Figure 1). This process ultimately results in the development of two independent and separate systems that will form the trachea and the esophagus (17).

The molecular processes that lead to compartmentalization, however, are not fully understood at present, and three main models have been proposed: the Outgrowth model (18, 19), the Watershed model (20), and the Septation model (21, 22).

The Septation model, which is currently the most accepted model of foregut development, suggests that lateral ridges of thickening epithelium, along the dorsoventral midline, make contact across the lumen and fuse together, forming the esophagotracheal septum. Subsequently, this septum moves rostrally to separate the trachea and esophagus (21). Definitive affirmation of this model has been hampered by the paucity of available data on the development of the lateral ridge.

According to the Outgrowth model, the trachea sprouts from the primitive foregut and elongates forming the respiratory tube from the larynx to the lungs, while the foregut itself differentiates into the esophagus (18, 19). By contrast, the Watershed model is based on the concept that a mesenchymal septum blocks elongation at the dorso/ventral midline of the foregut, while both trachea and esophagus elongate on the side (20). However, these two models are not supported by any scientific data. Both models postulate the presence of regions of increased proliferation, which has not yet been proven. For example, in the first scenario, a proliferation “hot-spot” would be expected where the trachea buds from the foregut. Furthermore, these models assume that the common foregut does not elongate while the compartmentalization takes place. Recent data however appear to suggest that the foregut tube actually decreases in length during the compartmentalization process (23). These findings taken together with genetic specification studies of the ventral foregut (24) lend weight to the Septation model.

## Molecular Specification of Foregut in Development and Implications for OA/TOF

**Figure 2:**
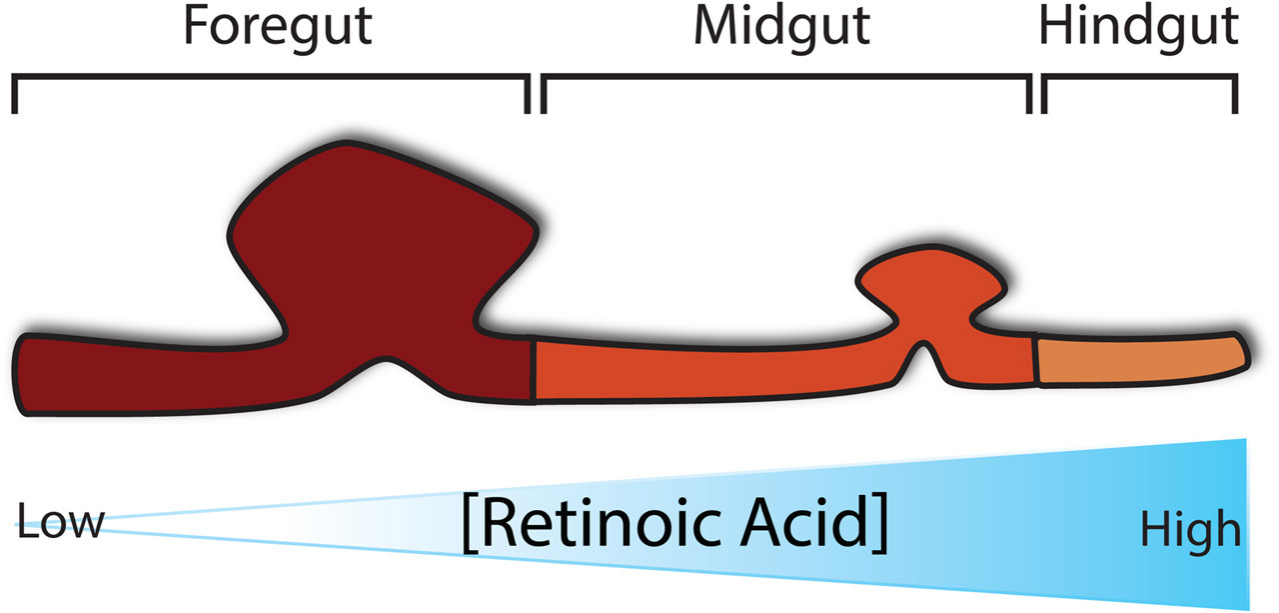
**Regional specification of the developing gut**. Specification of the developing gut is determined initially by a concentration gradient of retinoic acid along the anterior–posterior axis.

During gut development, many molecular pathways control and determine its regional specification. Of critical importance to the establishment of regional specification is the presence of retinoic acid (RA), a derivative of Vitamin A, along the anterior–posterior axis in a concentration-dependent manner, whereby the pharynx is exposed to little RA and the colon to highest concentration of RA (Figure 2) (25). This RA gradient induces the expression of various transcription factors in different regions along the gut tube, thus specifying each region in turn. Despite the fact that fetal vitamin A deficiency in humans has not been associated with OA/TOF, it has been reported that mice deficient in RA signaling develop foregut compartmentalization defects (26–28). In particular, the absence of retinoic acid receptors, specifically in mice lacking either all RARA isoforms and RARB2 or all RARB isoforms and RARA1, seems to block the foregut compartmentalization process, leading to the development of an undivided foregut with respiratory epithelium (28, 29). The role of RA in foregut development, along with its importance for pancreatic specification (30), has also been implicated in a mouse model that lacks RA-synthesizing retinaldehyde dehydrogenase 2 (*Aldh1a2*), which results in embryonic death at around E10.5. These mice, if rescued with a short dose of RA, reach birth but develop similar foregut defects together with other cardiovascular anomalies (31). In terms of foregut development, *Sox2* and *Pdx1* expression appear to be vital signaling components for specification of the esophagus and of the stomach and pancreas, respectively (30, 32, 33).

**Figure 3:**
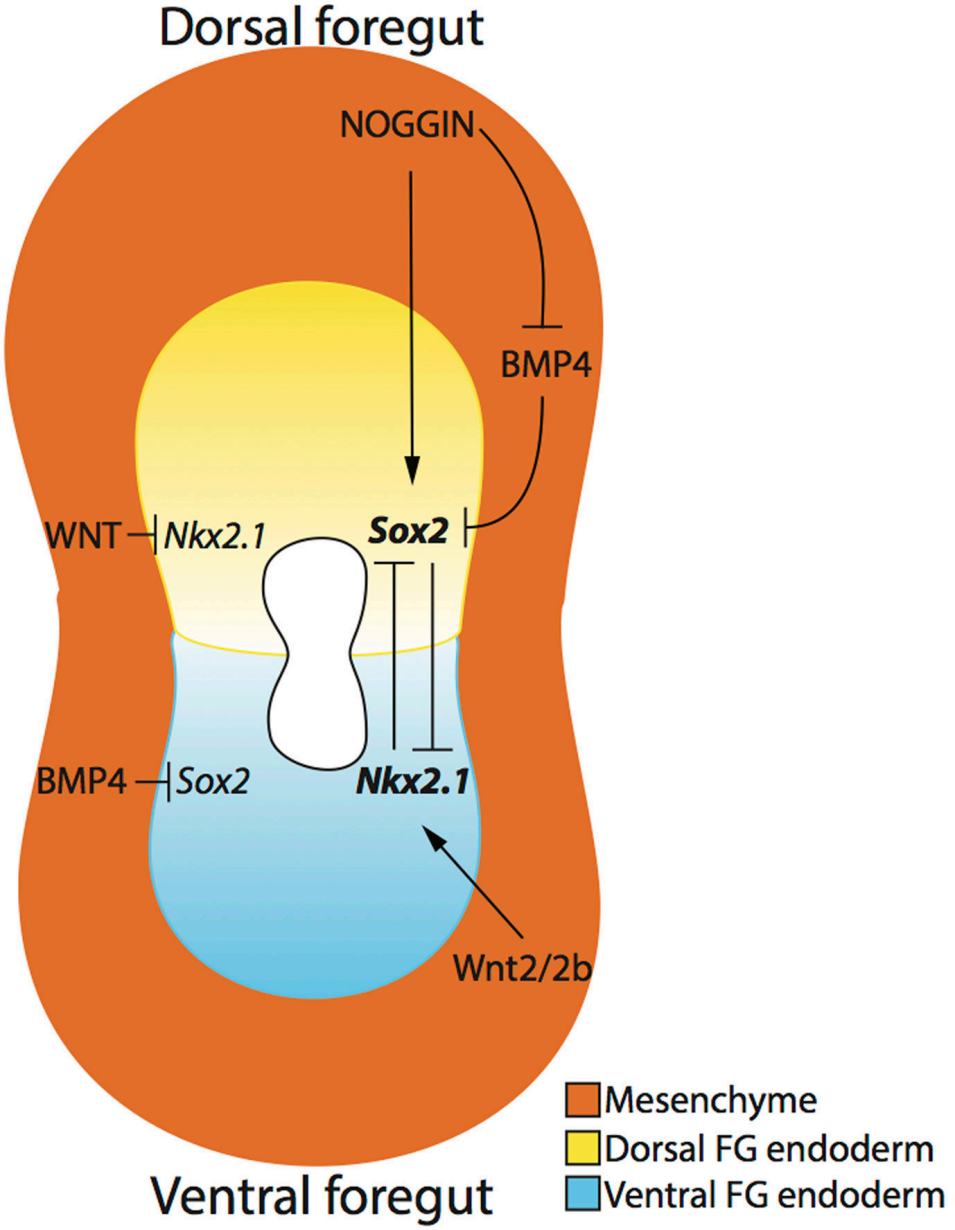
**Dorsoventral patterning of the developing foregut endoderm**. The dorsal (yellow) and ventral (blue) endoderm express *Sox2* and *Nkx2.1*, respectively. NOGGIN, produced by the surrounding mesenchyme (orange), regulates the expression of *Sox2* in the dorsal foregut endoderm by directly activating *Sox2* expression and indirectly inhibiting BMP4, which in turn inhibits *Sox2*. Ventrally, Wnt2/2b signaling activates the expression of *Nkx2.1* in the ventral foregut endoderm, and WNT signaling also inhibits *Nkx2.1* expression in the dorsal foregut endoderm. The mutual inhibition activity of *Nkx2.1* and *Sox2* create an expression gradient of these two genes, thereby allowing the separation of the two organs.

In addition, dorsoventral specification, at the molecular level, in the foregut endoderm may help explain how the compartmentalization process of the trachea and esophagus occurs (Figure 3). Specifically, the dorsal foregut endoderm expressing Sox2 gives rise to the esophagus, while the ventral foregut endoderm expressing the transcription factor Nkx2.1 (34) forms the trachea. Both *Sox2* and *Nkx2.1* seem to be crucial factors involved in foregut separation as revealed in transgenic mouse models. *Nkx2.1* null mice display incomplete foregut compartmentalization, resulting in a condition similar to tracheal agenesis with the lungs directly connected with the foregut, ultimately resulting in respiratory failure (34). The exact role of *Sox2* has been more difficult to determine as complete *Sox2* loss-of-function results in embryonic death pre-gastrulation (35). However, investigations using hypomorphic and null alleles of *Sox2* demonstrate that reduction in *S*ox*2* levels results in an OA with TOF phenotype 60% of the time (36). Moreover, this TOF phenotype displays respiratory characteristics, such as endodermal expression of *Nkx2.1* and the presence of cartilage (36). Therefore, it is clear that these two genes are necessary for organ specification of trachea and esophagus, but their specific role in the compartmentalization process is not proven (37).

Several signaling pathways determine the dorso/ventral patterning of *Sox2* and *Nkx2.1*. On the ventral side of the foregut, NKX2.1 protein expression is established by the production of BMP4 from the surrounding ventral mesenchyme, which acts through the BMP receptors BMPR1a, b in the ventral endoderm. If BMP4 is not produced in the mesenchyme or the endodermal receptors BMPR1a, b are absent, respiratory determination of the foregut will not proceed and tracheal agenesis may occur (38, 39). In this situation, *Sox2* expression appears to expand along the ventral aspect of the foregut, suggesting that BMP signaling is important for repressing ventral *Sox2* expression (37). Using a conditional knockout model of *Bmpr1a, b*, Domyan et al. demonstrated that subsequent suppression of *Sox2* can rescue *Nkx2.1* expression and the tracheal agenesis phenotype, suggesting that BMP signaling does not play a role in *Nkx2.1* specification, but rather in *Sox2* repression (38).

The BMP pathway is also important for dorsal foregut endoderm determination. More specifically, BMP ligands, produced in the ventral foregut mesenchyme, are counterbalanced by a BMP antagonist, NOGGIN, secreted by the dorsal foregut mesenchyme and the notochord (37). NOGGIN binds BMP4 to suppress BMP signaling in the dorsal endoderm (40), therefore allowing the expression of SOX2. Indeed, reduction in BMP antagonism causes OA/TOF as demonstrated by a 75% incidence of OA/TOF in *Noggin* null mutant mice (41). However, Fausett et al. have shown that *Noggin* is not critical for the dorso/ventral patterning of the foregut, which will express *Sox2* and *Nkx2.1* in the absence of *Noggin* as demonstrated *via* investigation of *Noggin* null mice (42).

The initial endodermal patterning of the foregut is subsequently stabilized by interactions between the endoderm and visceral mesoderm adjacent to the gut tube. This interaction is initiated by sonic hedgehog (SHH), a member of the Hedgehog family of morphogens expressed by the endoderm along the length of the gut (43, 44), which subsequently upregulates various transcription factors that are regionally expressed in the visceral mesoderm. These include homeobox-containing transcription factors (*Hox* genes) that are crucial for the morphogenesis and cytodifferentiation that determines structure along the length of the GI tract (44).

Shh ligand acts *via* binding to its receptor and through GLI1, 2, and 3 activating the transcription of target genes (45). For this reason, any deficiency in the downstream SHH pathway can cause disruption, mild to severe, in foregut development. For example, *Gli2* null mice do not exhibit severe problems, with only mild lung defects and hypoplastic trachea and esophagus. By contrast, *Gli2^−/−^;Gli3^+/−^* mice present with a more severe lung phenotype including delayed or incomplete separation of the trachea and esophagus (46). Moreover, hedgehog signaling seems to be critical in foregut compartmentalization as demonstrated by the development of abnormal esophageal and tracheal phenotypes in *Shh* null mice. In these mice, under-developed lung buds emerge directly from a single foregut tube connected to the stomach (43).

Another important molecular pathway involved in foregut specification is the WNT/*β*-catenin signaling pathway. WNT/*β*-catenin signaling has been proven to be necessary and sufficient for respiratory cell fate of the ventral foregut, provided that *Sox2* expression is repressed by BMP signaling as discussed previously (38). WNTs are secreted glycoproteins that act trough *β*-catenin (*Ctnnb1*), a cytoplasmic protein that translocates to the nucleus and binds transcriptional repressors ultimately inducing transcription of target genes (47). In terms of foregut development, WNT appears to be necessary for the ventral expression of *Nkx2.1*, with WNT2 and WNT2B, expressed in the ventral foregut mesenchyme, acting as important ligands involved in the compartmentalization process (48). Similar to loss of endodermal receptors BMPR1a, b in the foregut ventral endoderm, mesenchymal loss of WNT2 and WNT2b leads to disrupted endodermal expression of *Nkx2.1* and results in disrupted tracheal formation (48). Similarly, conditional deletion of the WNT signaling mediator *β*-catenin in mouse foregut mesenchyme and epithelium impedes the compartmentalization of the foregut, resulting in tracheal agenesis (39, 49). Conversely, a significant expansion in *Nkx2.1* expression through foregut endoderm, including the upper stomach epithelium, occurs if *Ctnnb1* is constitutively activated (48) further confirming the importance of WNT/*β*-catenin signaling.

In addition to the advances in knowledge achieved using transgenic approaches in various animal models, pharmacological studies using Adriamycin administration (50, 51) have provided additional means to study and analyze disruption in foregut development. Adriamycin, also called doxorubicin, is an anthracycline antibiotic and chemotherapeutic agent that, when injected in pregnant wild-type mice or rats before foregut compartmentalization, causes phenotypes of VACTERL syndrome (21, 24). Doxorubicin acts by interfering with replication and therefore inhibits DNA and RNA synthesis, in this way affecting multiple tissues and organs. Furthermore, doxorubicin also affects the SHH–GLI receptor signaling pathway, giving rise to abnormalities during foregut development as previously described (52). Due to the clinical association of OA/TOF with syndromic malformations such as VACTERL, CHARGE (coloboma, heart defects, atresia of choanae, retardation of growth, and ear abnormalities), and Di George Syndrome, other genetic traits have been investigated for possible association of foregut malformation. T-box genes are a family of transcription factors richly expressed in tissues undergoing active embryonic induction of organogenesis. *TBX1* has been shown to be a major determinant in 22q11 deletion syndromes (22q11DS), including Di George syndrome; hence, the influence of *TBX* gene activity in the developing foregut has recently attracted significant interest. Using both wild-type mice and the aforementioned Adriamycin model, McLaughlin et al. have demonstrated a focal pattern of *Tbx1* gene expression confined to the dorsal and ventral poles of the proximal wild-type esophagus. Altered *Tbx1* foregut expression in Adriamycin treated animals in this study further suggests that *Tbx1* may modulate normal esophageal development (53). Additional *Tbx* genes have been shown to play a role in foregut development. *Tbx4* expression has been demonstrated in the lung buds and mesenchyme surrounding the trachea (54). Furthermore, *Tbx4* has been shown to be specifically expressed in the visceral mesoderm of the developing lung in the chick model, and *Tbx4* misexpression shown to induce disrupted formation of the tracheo-esophageal septum, ectopic budding of the lung and TOF, further confirming the crucial involvement of *Tbx* gene activity in foregut embryology.

## Disruption of the Enteric Nervous System (ENS) in OA

Esophageal dysmotility is a very common and well-recognized disorder in children suffering OA (55). Kirkpatrick et al. reported uncoordinated contractile waves in the distal esophagus in 14 patients with OA (56), and others have associated GER with complications due to the surgical procedure, such as excessive tension on the vagus nerve or overt injury to it at the site of the esophageal anastomosis (57, 58). Although esophageal body motility dysfunction has been reported in patients following surgery, Lemoine et al., using high-resolution esophageal manometry before surgical repair in two children with isolated TOF, demonstrated that both had abnormal esophageal motility (hypomotility with distal contraction and complete aperistalsis) (59), suggesting that esophageal dysmotility is likely to be congenital.

This dysmotility is likely to be explained by loss, disruption, and/or dysfunction of the intrinsic innervation (ENS) of the esophagus. The ENS is derived principally from a population of vagal neural crest cells, which enter the foregut in humans at approximately week 4 (E9.5 in the mouse) (60) and migrate in a rostrocaudal fashion starting from the presumptive esophagus to colonize the entire gut by approximately week 7 (E13.5 in the mouse) (61, 62). To enable full gut colonization during embryogenesis, the neural crest cell population displays significant proliferative capacity. This proliferative capacity is tightly coordinated by *Ret*/GDNF signaling (63), while SOX10 and endothelin 3 signaling have been shown to be critical in the maintenance of multilineage ENS progenitors (64). The ENS is organized into two concentric plexuses, the inner submucosal plexus is present in the submucosa and an outer myenteric plexus is present between the circular and longitudinal muscle layers along the length of the GI tract. In the normal esophagus, the ENS is largely present in the myenteric plexus and the submucosal plexus is absent or sparsely present. Nakazato et al. showed that the myenteric (Auerbach) plexus of infants with OA is deficient. Specifically, a lower amount of neural tissue was present in the distal esophagus compared to the proximal end of untreated OA patients and control patients (65). More recently, Boleken et al. suggested that the expression of neuronal markers, such as neurofilaments, specifically found in neuronal cells, and synaptophysin, a calcium-binding protein present in the presynaptic vesicles of neurons, were significantly reduced in the affected part of the esophagus while S100 expression, a marker of glial cells, was increased in the muscular layers and the myenteric plexus (66). Of interest, GDNF expression, an important neurotrophic factor for neural cells, was significantly reduced in these OA patients, suggesting a possible signaling deficiency, which could account for the observed intrinsic innervation deficits (66).

## The Role of Stem Cell Therapy and Tissue Engineering in the Treatment of OA/TOF

Despite advances in our understanding of the genetic determinants of foregut development, this knowledge has not translated, as yet, to improved therapeutic interventions in the treatment of OA/TOF. Hence, alternative approaches using novel techniques such as gene and stem cell therapy in combination with advancing tissue engineering protocols may provide alternative routes for treatment of these difficult disorders following standard surgical intervention and pharmacological management. The current limitations of surgical approaches for the treatment of OA and TOF combined with the ongoing post-operative symptoms experienced by patients have provided the impetus to investigate potential cell-based therapies alone or combined with tissue engineering as a means of replenishing missing or dysfunctional cell types or indeed absent sections of esophagus. Alternatively, they may provide a mechanism to treat ongoing foregut dysfunction, post-surgery, in less severe cases.

Arguably, the most promising approach lies in esophageal tissue engineering as a potential replacement of tissue segments. Tissue engineering approaches, using acellular scaffolds derived from animals and humans, or cell-seeded grafts, have recently been investigated (67). In particular, similar to a previous report for the trachea (68), decellularized esophageal scaffolds have been used with good results in both preclinical and clinical studies (69). Significant heterogeneity exists among studies, both with respect to the type of scaffold, and extent of surgery and species used, which partly explains the range of results reported. Badylak et al. laid sheets of small intestinal submucosa (SIS) onto the raw internal surface of the esophagus following endoscopic submucosal resection in five patients with superficial cancers (69). The scaffold promoted physiological remodeling and decreased the chance of stricture formation. Moreover, a commercially available extracellular matrix was able to promote full-thickness regeneration of the esophagus with stratified squamous epithelium, a normal five-layer wall, and peristaltic motility with bolus transit (70). Decellularized esophageal tissue retains signals, both chemical and structural, which should promote appropriate migration and differentiation of host cells (71–73), which may be unlikely to occur with scaffolds originating outside the esophagus, such as SIS. In an attempt to engineer a complex structure more closely resembling normal esophagus, Nakase et al. developed an elegant method for producing an esophageal construct. Oral keratinocytes and fibroblasts were cultured on human amniotic membrane and smooth muscle cells cultured on PGA. The two layers were rolled into a tube, implanted in the omentum, harvested at 3 weeks and used to replace a partial defect (74). Similarly, circumferential replacement of the cervical esophagus was achieved using a tube-shaped tissue-engineered acellular substitute with autologous skeletal myoblasts covered by a human amniotic membrane seeded with autologous oral epithelial cells. Under the temporary cover of an esophageal endoprothesis, which was removed at 6 months, animals were able to reach nutritional autonomy and at sacrifice the tissue remodeled toward an esophageal phenotype (75).

While significant steps have been made in the ability to expand both epithelial and muscle cells for tissue engineering purposes (68), it will be essential to neo-innervate any potential engineered scaffold to allow for full restoration of function. To this end, major strides have been made in the last decade in the identification and isolation of enteric neural stem cells (ENSCs), which may not only provide an ideal candidate for neo-innervation of tissue-engineered scaffolds but may also provide a mechanism of restoring function in patients where ongoing dysfunction, following surgery, is found to be neuropathic. A number of studies have demonstrated that the human postnatal GI tract contains multipotent cells that upon transplantation can colonize the gut and differentiate into appropriate enteric neural phenotypes (76, 77). The proliferative capacity and multipotent nature of these neural crest derivatives has lead to investigation of the identification and isolation of ENSCs. Recent investigations have sought to utilize such ENSC as a means of replacing lost or absent neurons in a number of GI disease models. Both mouse and human ENSC have been shown to integrate within mouse colonic tissues after transplantation (78–80). Previous studies have also demonstrated that ENSC can colonize aneural colonic tissues *ex vivo* (77). Importantly, both embryonic and postnatal mouse ENSC have been shown to integrate, differentiate into appropriate neuronal subtypes, and form functional neurons *in vivo* in recipient mouse models where the endogenous ENS persists (79). Furthermore, it has more recently been shown that human ENSC have the ability to colonize gut and integrate with the endogenous ENS in wild-type mouse colon, including functional integration of human fetal ENSC (78). These studies provide critical evidence that ENSC may provide a mechanism to restore function in various gut tissues. Significantly, ENSCs have been identified in both human fetal (78) and postnatal tissues (77, 81), demonstrating the possibility of an autologous source of neural stem cells which could be harvested relatively easily *via* endoscopy, from other bowel regions, expanded, and then transplanted *via* tissue-engineered scaffolds or autologous transplantation directly to the esophagus. A significant advantage of this approach would be the ability to circumvent immunological rejection of autologously transplanted cells. It may also be possible to perform heterologous transplantation of ENSC from matched donors; however, such an application is likely undesirable due to the possible requirement of lifelong immunosuppression. Future studies including preclinical evaluation of the ability of ENSC to provide functional rescue of foregut disorders and provide functional innervation within tissue-engineered specimens are required, prior to implementation of any clinical trials in human patients. One significant caveat regarding the use of ENSC is the potential limitation in their expansion characteristics. Transplantation studies, to date, have demonstrated relatively modest expansion and integration of ENSC within transplanted colonic tissues (79, 80), which may impact on their ability to restore function in large-scale human tissues. It remains possible that significant cell numbers will be required for the treatment of OA; therefore, studies of alternative cell sources are additionally required to determine the best cellular source for esophageal neo-innervation.

To this end, there has been significant interest in the potential use of pluripotent stem cell (PSC) populations as a source of regenerative neural cells. Both embryonic stem (ES) cells and induced pluripotent stem (iPS) cells have the capacity to give rise to any cell of the body. Both mouse and human pluripotent stem cells (ES and iPS cells) can be differentiated into “ENS-like” cells (82) with capacity to proliferate limitlessly and therefore may provide an ideal cellular source for neo-innervation studies.

Of particular interest, recent studies have shown that ES and iPS cells can be manipulated *in vitro* to induce a neural crest-like phenotype (83–85). Recent work has demonstrated that human iPS-derived vagal-like neural crest cells can be combined with human pluripotent stem cell-derived intestinal organoids to form functional organoid units complete with neuronal reflexes (83). The ability to source autologous patient-derived iPS cells, which can be subsequently driven toward and ENS phenotype may revolutionize treatments for enteric neuropathies allowing autologous cell therapy without lifelong immunosuppression. However, at present, limited data exist as to their integration and the ability of such cells to functionally rescue gut motility. Interestingly, Fattahi et al. recently suggested that human ES- and iPS-derived enteric neural crest could rescue a mouse model of Hirschsprung disease after *in vivo* transplantation. Transplantation of these human-derived vagal neural crest cells to the colon of EDNRB^s-l/s-l^ (SSL/LEJ) mice led to 100% survival; however, no mechanisms regarding the integration of these cells within the host neuromusculature, or the functional rescue achieved at the organ level, were presented (84). Therefore, further work is crucially required to establish the functional integration of PSC-derived neural crest cells after *in vivo* transplantation in a number of model systems.

While the potential expansion and manipulation of pluripotent stem cells provide an exciting proposition above that of ENSC, several issues remain to be addressed prior to their validation as a suitable treatment option. One critical issue regarding the potential use of pluripotent stem cell sources is the potential introduction of residual pluripotent stem cells, which could be tumorigenic. Furthermore, studies are required to both consolidate and standardize protocols for the derivation of pure enteric neural crest cells and establish safety parameters for such pluripotent protocols, including genetic and epigenetic stability given that such derivations usually require significant culture periods. Such studies will allow for critical determination of the beneficial impacts of these cell replacement sources above that of autologously sourced ENSC.

## Conclusion and Future Directions

The management of esophageal atresia remains challenging. This stems in part from a failure to understand the precise molecular mechanisms that underlie normal foregut development and the aberrations that lead to disease such as OA. As a result, therapies for OA are limited and designed to palliate rather than cure. Even when primary anastomosis is achieved in OA, the esophagus is often dysfunctional leading to major gastric and respiratory problems associated with poor quality of life. Treatments of complications related to OA are unsatisfactory and may require multiple surgeries. Some strides toward a better understanding of normal and abnormal development of the foregut have been made, but there is still a need for focused research in this area. This could lead to the development of innovative treatments. Regenerative medicine may have a role not only for *filling the gap* when primary anastomosis is not possible but also for ameliorating esophageal dysfunction. Alternatively, such dysfunction may be addressed more simply and directly utilizing the significant advances that have occurred in the field of ENSC biology. The transplantation of such cells may provide an adjunct to surgery to improve outcomes. Either way, the coming decade may well herald exciting prospects for the understanding of the origins of OA and the development of definitive therapies.

## Author Contributions

NT conceived the work. SP and CM contributed equally to the work and are joint first authors. SP designed and made the figures and together with CM wrote the first draft. NT, OB, and PC helped draft the final manuscript and revised it critically for important intellectual content. All authors critically reviewed the final manuscript.

## Conflict of Interest Statement

The authors declare that the research was conducted in the absence of any commercial or financial relationships that could be construed as a potential conflict of interest.
